# Age-dependent and coordinated shift in performance between implicit and explicit skill learning

**DOI:** 10.3389/fncom.2013.00147

**Published:** 2013-10-22

**Authors:** Dezso Nemeth, Karolina Janacsek, József Fiser

**Affiliations:** ^1^Memory and Language Lab, Department of Clinical Psychology and Addiction, Eötvös Loránd UniversityBudapest, Hungary; ^2^Department of Cognitive Science, Central European UniversityBudapest, Hungary

**Keywords:** probabilistic sequence learning, associative learning, development, model-based vs. model free learning

## Abstract

It has been reported recently that while general sequence learning across ages conforms to the typical inverted-U shape pattern, with best performance in early adulthood, surprisingly, the basic ability of picking up in an implicit manner triplets that occur with high vs. low probability in the sequence is best before 12 years of age and it significantly weakens afterwards. Based on these findings, it has been hypothesized that the cognitively controlled processes coming online at around 12 are useful for more targeted explicit learning at the cost of becoming relatively less sensitive to raw probabilities of events. To test this hypothesis, we collected data in a sequence learning task using probabilistic sequences in five age groups from 11 to 39 years of age (*N* = 288), replicating the original implicit learning paradigm in an explicit task setting where subjects were guided to find repeating sequences. We found that in contrast to the implicit results, performance with the high- vs. low-probability triplets was at the same level in all age groups when subjects sought patterns in the sequence explicitly. Importantly, measurements of explicit knowledge about the identity of the sequences revealed a significant increase in ability to explicitly access the true sequences exactly around the age where the earlier study found the significant drop in ability to learn implicitly raw probabilities. These findings support the conjecture that the gradually increasing involvement of more complex internal models optimizes our skill learning abilities by compensating for the performance loss due to down-weighting the raw probabilities of the sensory input, while expanding our ability to acquire more sophisticated skills.

## Introduction

In order to fully understand the mechanism of complex skill acquisition, the defining characteristics of both explicit and implicit learning, such as their efficiency across life span, and their interaction must be clarified. Sequence learning is a prominent component of skill learning, which is involved in obtaining not only motor, but also cognitive and social skills. It is ideally suited to investigate, in a controlled way, the interplay between the fundamental mechanisms defining implicit/automatic as well as explicit learning. In the present study, we used a sequential learning paradigm to explore the developmental interaction between human explicit and implicit learning.

Although there are various proposals regarding the age-related developmental changes in late adulthood based on changes in working memory capacity, response selection demands, or the spatial requirement of the task (see Bo and Seidler, 2010; Bo et al., 2012; Janacsek and Nemeth, 2013), in the development from childhood to adulthood, there are three major proposals about the development of sequence learning in humans. The first posits that there is no significant change with age in the ability of learning sequences implicitly, in other words sequence-learning is age-invariant (Meulemans et al., 1998; Vinter and Perruchet, 2000). According to a second proposal, the developmental pattern of sequence learning across ages conforms to the typical inverted-U shape pattern, with best performance at the age of mid-20s (Maybery et al., 1995; Fletcher et al., 2000; Thomas et al., 2004) corroborating the traditional view of a steady improvement of general cognitive learning abilities until well into adulthood (Craik and Bialystok, 2006). The third proposal is based on the surprising finding that, the basic ability of picking up statistical properties of a presented sequence in an implicit manner is best before 12 years of age and it significantly weakens afterwards as measured by the raw RT difference between the high and low frequency triplets found in a probabilistic sequence learning task (Janacsek et al., 2012). The results of this study implied a marked decrease in this sensitivity around the age of 12, which is in contrast to both earlier proposals. It is important to notice that contrary to the studies of the previous two proposals, the last study is based not on a deterministic but on a probabilistic sequence learning task, which can measure finer, computationally relevant aspects of the learning process.

Specifically, the Janacsek et al. (2012) study proposed that this discrepancy with classical results might be explained by a shift in the structural development of implicit learning based on two lines of evidence. First, although the raw probabilities of the sensory environment are important for learning and both infants (Saffran et al., 1996, 1999; Aslin et al., 1998; Fiser and Aslin, 2002) and adults (Fiser and Aslin, 2001; Hunt and Aslin, 2001) are highly sensitive to these probabilities, there is an ongoing debate on how using these simple probabilities can lead to a highly complex knowledge of the world, such as sensory invariances and development of a language (Gomez and Gerken, 1999; Marcus et al., 1999; Nemeth et al., 2011). Recent studies proposed that using an internally stored structured model of the world that emerges based on past experience together with probabilistic learning could help to address this issue and also provide evidence that humans might implement such a strategy during implicit learning (Orban et al., 2008; Tenenbaum et al., 2011). In this framework, as the internal model develops, past experiences become more influential, and therefore, internal interpretations of events become more elaborate and less directly related to their raw occurrence probabilities experienced momentarily. There is ample evidence for both internal model dependent and independent learning in human and animals (Packard and Knowlton, 2002; O'Doherty et al., 2004), and a recent study argued that from a normative standpoint, existence of such multiple learning mechanisms in the brain (cf. model-free vs. model-based learning) with an uncertainty–based arbitration between them would be computationally optimal (Daw et al., 2005). Anchoring this hypothesis biologically, it has been suggested that the presumed mechanisms related to model-free and model-based learning were related to the basal ganglia vs. the prefrontal areas and temporal lobe of the cortex, respectively (Daw et al., 2005).

The second line of evidence provides support for the separated, complementary, and also competitive nature of the prefrontal- and medial temporal lobe (MTL)-dependent learning based on internal models vs. basal ganglia-dependent model-free learning. Various studies investigating learning under specific conditions showed that obstructing the PFC and/or MTL by a demanding secondary task (Foerde et al., 2006) do not adversely affect implicit learning. Other studies found that inserting a task between the learning sessions (Brown and Robertson, 2007a,b), performing a working memory and an implicit learning task simultaneously (Filoteo et al., 2010), or a neuropharmacological blockage (Frank et al., 2006) even had a positive effect on performance in an implicit learning task. Moreover, a recent study found a 3-fold boost of implicit statistical sequence learning by hypnosis, presumably caused by the disconnection of the frontal lobe from other brain areas, reducing the competition between brain systems (Nemeth et al., 2013a). Importantly, it is known that the cortical areas connected to the internal models related to model-based learning become truly functional late in the development, around the age of 12 (Giedd et al., 1999; Blakemore and Choudhury, 2006), which is about the age at which Janacsek et al. (2012) found the sudden decrement in sensitivity to the relative raw probabilities.

Based on these two lines of evidence, Janacsek et al. (2012) proposed that the emerging functionality at around 12 signals the shift when the system adapts efficiently to more complex aspects of the world by relying more on internal model-based interpretations, while somewhat neglecting the raw probabilities of the sensory input, and therefore, decreasing the ability to develop and stabilize fundamentally new basic competences. Thus in fact, the seemingly paradoxical result of gradually becoming less sensitive to basic statistics, if timed appropriately, could be the optimal strategy for human skill learning in general.

The Alternating Serial Reaction Time (ASRT) Task (Howard and Howard, 1997) is a unique tool to investigate the computational background of this conjecture, because we can measure different processes, which are related more to internal model building or more to model-free learning in the same experimental design. In the ASRT task, participants are asked to respond to stimuli, which appear according to a probabilistic sequence structure (e.g., 2r1r3r4r, where numbers represent specific locations on the screen determined by the sequence, and r represent randomly selected location). Because of this probabilistic structure, we can determine several different or partly different learning measures: triplet learning, statistical learning, higher-order sequence learning, and maximized learning (Howard and Howard, 1997) (see method part). From the point of view of model-free and model-based learning the two prominent types of learning are (1) *Statistical Learning* defined as the differentiation between high and low frequency elements only in randomly appearing stimuli, which makes it possible to measure purely frequency-based learning, and (2) *Higher-order sequence learning* defined as the differentiation between elements appearing in a larger sequential pattern vs. appearing randomly when the appearance frequencies of these elements are controlled. Thus *statistical learning* does not require previously built-up representation beyond the detection of relative frequencies of simple repetitive events leading more easily to a model-free type of learning. In contrast, *Higher-order sequence learning* must be based on a more global and complex representation of sequence structure defined by interactions of multiple events one experiences across space and time and therefore, it is related more to model-based processes.

To sum up, it has been hypothesized by Janacsek et al. (2012) that the cognitively controlled processes coming online at around 12 are useful for more targeted explicit learning at the cost of becoming relatively less sensitive to raw probabilities of events. To test this hypothesis, we collected data in an ASRT sequence learning task using probabilistic sequences in five age groups from 11 to 39 years of age, replicating the original implicit learning paradigm in an explicit task setting, where participants were guided to find repeating sequences, and compared it to the original implicit learning task. With the help of this experimental design, we could draw the developmental differences separately for statistical learning of raw probabilities and for more complex, higher-order sequence learning. Moreover, by analyzing the course of learning across the task in more detail, we were able to characterize the development of model-based processes across ages and conditions (explicit vs. implicit) more specifically.

## Methods

### Participants

There were 288 participants in the experiment, between the ages of 11 and 39, that were clustered into five age groups between 11–13, 14–15, 16–18, 19–29, and 30–39 years of age (Table 1). Half of the participants took part in the explicit condition and half in the implicit condition [some results of the latter data were already published in the paper of Janacsek et al. (2012)]. None of the participants suffered from any developmental, psychiatric, or neurological disorders. All participants gave signed informed consent (parental consent was obtained for children) and received no financial compensation for participation. The study was approved by the National Psychological Ethical Committee of Hungary.

**Table 1:** **Demographic data and mean RT in the different groups**.

**Condition**	**Age group**	**Age**	**Sex**	**Education**
Explicit	11–13-years-old (*n* = 23)	11.35 (0.71)	11 M/12 F	5.13 (0.34)
	14–15-years-old (*n* = 23)	14.87 (0.34)	12 M/11 F	7.91 (0.29)
	16–18-years-old (*n* = 38)	17.00 (0.40)	13 M/25 F	10.63 (0.67)
	19–29-years-old (*n* = 43)	21.30 (2.02)	26 M/17 F	14.49 (1.74)
	30–39-years-old (*n* = 20)	35.10 (3.21)	11 M/9 F	15.55 (2.42)
Implicit	11–13-years-old (*n* = 24)	11.58 (0.65)	16 M/8 F	4.64 (0.73)
	14–15-years-old (*n* = 21)	14.71 (0.46)	13 M/8 F	7.95 (0.67)
	16–18-years-old (*n* = 24)	17.04 (0.36)	12 M/12 F	10.45 (0.52)
	19–29-years-old (*n* = 45)	21.71 (3.01)	29 M/16 F	14.98 (2.42)
	30–39-years-old (*n* = 27)	34.78 (2.21)	14 M/13 F	17.44 (3.53)

In all columns, numbers in parentheses show standard deviation.

### Task and procedure

**Figure 1:**
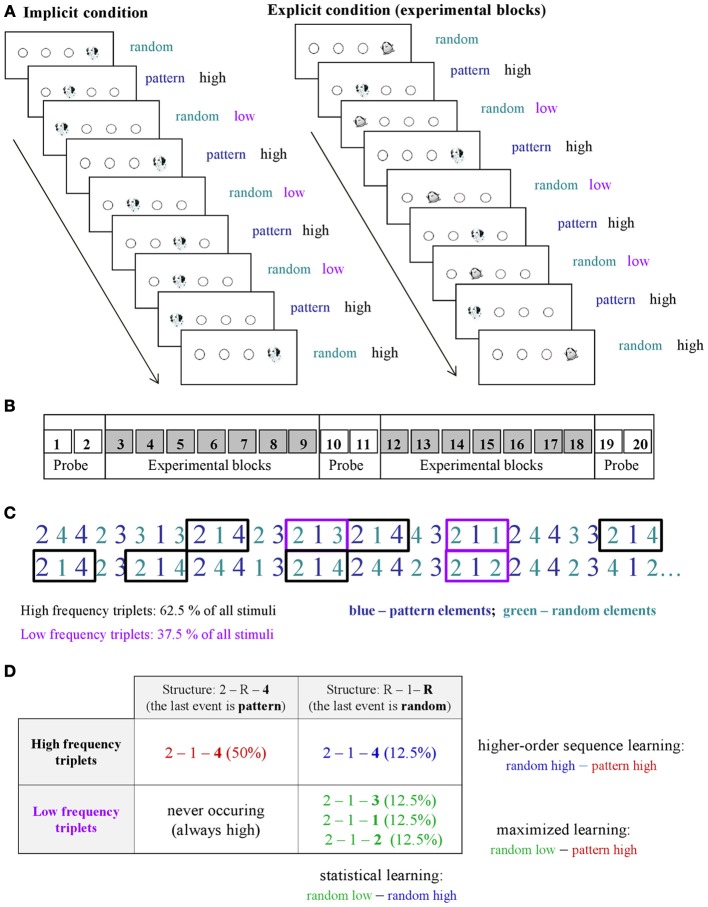
**Design and learning measures in the study. (A)** An implicit and an explicit version of the ASRT task were administered in the experiment. In the explicit version of the task (right panel), the regularity was marked by using different stimuli for sequence elements (a dog's head) and for random ones (penguin). In the implicit condition (left panel), sequence and random elements were not marked differently (a dog's head was used always). **(B)** There was a total of 20 blocks in the study: Block 1–2, 10–11, and 19–20 were called “probe blocks” in which all sequence elements were marked with the same picture (a dog's head), while the underlying structure of the sequence was the same as in the remaining blocks, “the experimental blocks” where an explicit marking denoted the random (penguin) and pattern elements (dog). **(C)** As the ASRT task contains an alternating sequence structure (e.g., 2r4r3r1r, where numbers correspond to the four locations on the screen and the r represents randomly chosen locations), some runs of three consecutive elements (called triplets) occur more frequently than others. For subsequent analyses, we determined for each stimulus whether it was the last element of a high-frequency triplet (black frames) or low-frequency triplet (purple frames). **(D)** We assessed *pure statistical learning* (see text) by comparing the responses for those random elements that were the last elements of a high frequency triplet, opposite to those that were the last of a low frequency triplet (right column). In contrast, *higher-order sequence learning* was assessed as a difference between responses for pattern elements (which are always high frequency triplets) vs. random-high frequency triplet elements (top row). The additive effect of statistical and higher-order sequence learning is called *maximized learning* in our study (upper left vs. lower right cells).

Learning was measured by the ASRT task (Howard and Howard, 1997). In this task, a stimulus (e.g., a dog's head; Figure 1A) appeared in one of four empty circles on the screen and participants had to press the corresponding button when it occurred. The computer was equipped with a special keyboard with four heightened keys (Y, C, B, and M on a Hungarian keyboard; equivalent to Z, C, B, M on a US keyboard), each corresponding to the circles in a horizontal arrangement. The task was presented in blocks with 85 stimuli: the first five button pressings were random for practice purposes, then an 8-element alternating sequence (e.g., 2r4r3r1r, where each number represents the one of the four circles on the screen and r represents a randomly selected circle) repeated ten times. The response to stimulus interval was 120 ms (Song et al., 2007a; Nemeth et al., 2010).

An implicit and an explicit version of the ASRT task were administered in the experiment. In the *implicit version* of the task, participants were informed that the main aim of the study was to find out just how extended practice affected performance on a simple reaction time task. Therefore we emphasized performing the task as fast and as accurately as they could. They were not given any information about the regularity that was embedded in the task (Nemeth et al., 2010). In the *explicit version* of the task, the regularity was marked by different stimuli for sequence and random elements (cued experimental blocks—Song et al., 2007b). In order to maintain the attention and motivation of the children we chose pictures of animals to indicate sequence (a dog's head) and random (a penguin) elements (Figure 1A). Participants were informed that penguin targets always had randomly chosen locations while dog targets always followed a predetermined pattern. They were instructed to find the hidden pattern defined by the dog heads in order to improve their performance, thus to be faster and more accurate using this sequence information to predict the sequence elements.

The ASRT consisted of 20 blocks. As one block took about 1–1.5 min, the task took approximately 20–30 min. In the explicit condition, Blocks 1–2, 10–11, and 19–20 were *probe blocks* (Figure 1B), where sequence and random elements were not indicated (dog's head was used for all stimuli). In these probe blocks participants were not told that there would be any regularity in the sequence, although the same regularity was included as the one in the cued blocks. Although our study focuses on experimental blocks, the main aim of inserting the probe blocks was to be able to compare the performance in implicit and explicit conditions more directly utilizing the fact that in these blocks neither group was informed about the regularity.

*Explicit knowledge* about the sequence was measured after each cued block in the explicit condition. Participants were instructed to report any regularity they noticed and the experimenter registered their answers. This method allowed us to determine the duration (in term of the number of blocks) participants needed to learn the sequence correctly as defined by consistently reporting the same sequence from that point on in the remaining blocks. In the implicit condition, participants were not asked to report the regularity after each block because this instruction would have made them focus on finding the regularity, thus it would eliminate the instruction differences between the two conditions. Rather, to determine the amount of explicit knowledge the participants acquired about the task in the implicit condition, a short questionnaire was administered after the experimental session (Song et al., 2007a). This questionnaire included increasingly specific questions, such as “Have you noticed anything special regarding the task?,” “Have you noticed some regularity in the sequence of stimuli?.” The experimenter rated subjects' answers on a 5-point scale where 1 denoted “Nothing noticed” and 5 denoted “Total awareness.” Importantly, none of the participants in the implicit condition, children or adult, reported noticing the hidden repeating sequence.

For each participant, one of the six unique permutations of the four possible ASRT sequence stimuli was selected in a pseudo-random manner, so that the six different sequences based on a permutation rule were used equally often across participants (Howard and Howard, 1997; Nemeth et al., 2010).

### The stimulus structure in the ASRT task

We will discuss two important aspects of the statistical structure defined by our ASRT sequences. We define *long-range correlations* to refer to all statistical dependencies due to correlations coming from adjacent and non-adjacent co-occurrences not between the elements of three consecutive locations in the sequence, i.e., triplet, but the element of the triplet and some preceding other elements. These correlations are strongly related to the predetermined sequences of the task. In addition, we define *local structures* as statistical relations coming from all other statistical regularities but not from the predetermined sequence structure.

Regarding the local sequence structures, in the alternating sequence structure of our ASRT task (e.g., 2r4r3r1r), some triplets (i.e., combinations of three consecutive events) occurred more frequently than others. Importantly, there are two different ways how such frequent triplets could occur. For example, in the above illustration, 2_4, 4_3, 3_1, and 1_2 (where “_” indicates the middle element of the triplet) occurred often, and they did so either by the third element (bold numbers) being derived from the sequence or so that it was a random element. In contrast, infrequent triplets could occur only in one way. Specifically, 1_3 or 4_1 triplets occurred less frequently only so that the third element was random (Figures 1C,D). Following previous studies, we refer to the former as *high-frequency triplets* and the latter as *low-frequency triplets*. Note that due to the higher occurrence probability, the final event of high-frequency triplets was more predictable from the initial event of the same triplet compared to the low-frequency triplets [also known as non-adjacent second-order dependency (Remillard, 2008)]. To quantitatively assess the effect of these differences in occurrence probabilities on learning, for each stimulus/event, we determined whether it was the last element of a high- or low-frequency triplet providing one independent factor of the learning process (Figure 1D).

The second aspect of the statistical structure of the ASRT sequences is defined by the long-range correlations, the dependencies beyond the triplet that are due to the four non-adjacent elements following a preset sequence. This effect can be quantified by noticing that triplets with the last element being “random” have strong correlations between the middle element of the triplet and the elements preceding the triplet. In contrast, triplets with “pattern” last element have such correlations only with elements further away from the beginning of the triplet. The effect of this difference in distance-dependent correlations on human performance is unknown. Nevertheless, the dichotomy between pattern- and random-last triplets provides the second independent factor in our design to understand what drives skill learning (columns of Figure 1D). To quantify the effects, first we have calculated the relative probabilities of these different triplet types and found that out of the 64 possible triplets in the task (4^3^, 4 stimuli combined for three consecutive events), 16 are high frequency triplets, each of them occurring in approximately 4% of the trials, about five times more often than the low-frequency triplets. Thus, approximately 62.5% of all trials are high-frequency triplets and the remaining 37.5% of trials are low-frequency ones, while out of the 62.5% of the high-frequency triplets 50 and 12.5% are pattern-last and random-last triplets, respectively (Figure 1D). Note, that each trial (i.e., presentation of a stimulus) is defined exclusively either as the last element of a high- or a low-frequency triplet based on the n-2 trial (Howard and Howard, 1997; Janacsek et al., 2012).

## Results

### Learning types in the ASRT task

Previous ASRT studies used several methods for analyzing learning in the ASRT task. The first option is to measure the overall difference between responses for pattern vs. random elements (pattern-random learning; e.g., Howard and Howard, 1997). However, this measure neglects the differences in probabilistic structure of the sequence based on 2-lag non-adjacent second-order dependencies, i.e., the fact that some triplets are more frequent than others. Since it is known that people are sensitive to such probabilistic nature of a sequence by being faster on more frequent triplets compared to the less frequent ones, more recent, studies also compared responses to high and low frequency triplets separately (triplet learning; e.g., Howard and Howard, 1997; Janacsek et al., 2012; Nemeth et al., 2013a). Note however, that this measure still collapses high frequency triplets across random-last and pattern-last triplets (compared the two rows of Figure 1D). Hence, knowledge about the sequence structure independent of the local statistical features—cannot be extracted from this learning measure alone. To overcome this problem, in some studies an additional learning measure was introduced based on the difference between responses for high frequency pattern-last and high-frequency random-last elements as measured between the two columns of the first row in Figure 1D (Howard and Howard, 1997; Song et al., 2007a,b). However, a systematic comparison of these measures and clarification of their relation within a single study has not been done before.

To dissect the various effects contributing to sequence learning, we used the measures above and added new statistical measures to assess the amount of pure statistical learning in the ASRT task. We define *pure statistical learning* as the difference in responses between high-frequency and low-frequency random-last triplets (right column, Figure 1D). In this case, the sequence properties are the same: both are random-last triplets (finishing with a penguin stimulus) the only difference between the two groups being statistical in nature: whether those triplets are more or less frequent. Thus, statistical learning is defined as faster responses for high frequency random elements compared to low frequency ones. Note, that statistical learning measures a different effect than higher-order sequence learning: the first assesses purely the benefit of presentation frequency differences of local elements, while the second one measures the effect of long-range repetitions due to the predetermined multi-element sequence. This means that assuming independence between these two measures, we should see an additive effect of these two types of learning when comparing responses for pattern elements vs. random low frequency elements (*maximized learning*, upper right vs. lower left cells in Figure 1D) with statistical and higher-order sequence learning results. We tested this hypothesis by calculating and comparing all these learning effects.

In our study, we first report the triplet learning results because this has been the most common analysis method in the ASRT studies and thus it gives us the opportunity to directly compare our results with those of previous studies. Next, we compare the developmental trajectory of statistical and higher-order sequence learning across implicit and explicit conditions between ages of 11 and 39 years, using the above mentioned measures to obtain a more detailed picture about the underlying mechanisms in probabilistic sequence learning tasks.

### Triplet learning across age groups and conditions

**Figure 2:**
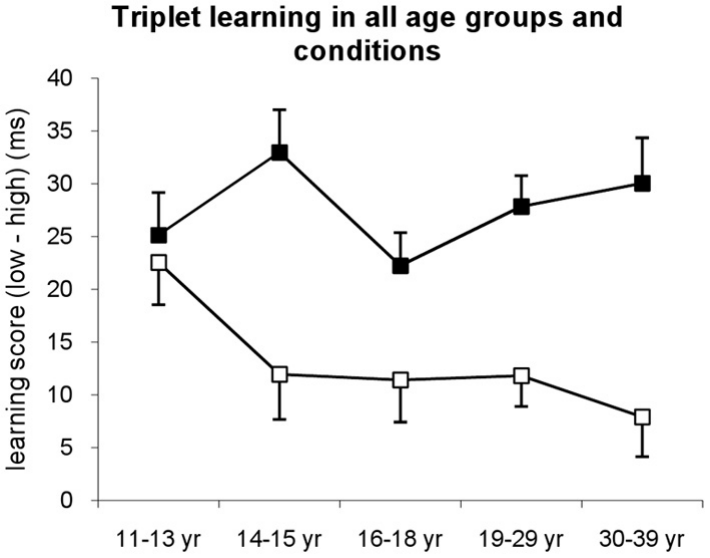
**Triplet learning in all age groups separately for explicit (filled squares) and implicit (open squares) conditions.** Learning score was defined as the difference between RTs for low vs. high frequency triplets. In the implicit condition, the 11–13-years-old group showed the highest learning differing from all other groups, while in the explicit condition all groups performed at the same level. Error bars represent standard error of mean (s.e.m.).

To compare triplet learning among age groups and conditions, first we conducted a mixed design ANOVA for the *experimental blocks* (as defined in Figure 1B) with TRIPLET (2: high vs. low frequency) and BLOCK (1–14) as within-subject factors, and AGE GROUP (11–13, 14–15, 16–18, 19–29, and 30–39 years of age) and CONDITION (explicit vs. implicit) as between-subjects factors (Figure 2). All significant results are reported together with the η^2^_*p*_ effect size and Greenhouse Geisser ε correction factors where applicable. Planned comparisons and *post-hoc* analyses were conducted by Fisher's LSD pairwise comparisons.

The ANOVA revealed significant triplet learning [indicated by the significant main effect of TRIPLET: *F*_(1, 278)_ = 291.55, η^2^_*p*_ = 0.51, *p* < 0.001] such that RTs were faster on high than on low frequency triplets. The conditions differed in the extent of this triplet learning [shown by the significant TRIPLET × CONDITION interaction: *F*_(1, 278)_ = 37.49, η^2^_*p*_ = 0.12, *p* < 0.001]: the participants in the explicit condition were 27.66 ms faster on high than on low frequency triplets, while this difference was only 13.12 ms in the implicit condition. Overall, age groups showed similar extent of learning [TRIPLET × AGE GROUP interaction: *F*_(1, 278)_ = 1.10, η^2^_*p*_ = 0.02, *p* = 0.357]; however, there was a trend in the TRIPLET × AGE GROUP × CONDITION interaction [*F*_(1, 278)_ = 2.00, η^2^_*p*_ = 0.03, *p* = 0.095], suggesting different learning performance across age groups in implicit vs. explicit conditions.

**Figure A1:**
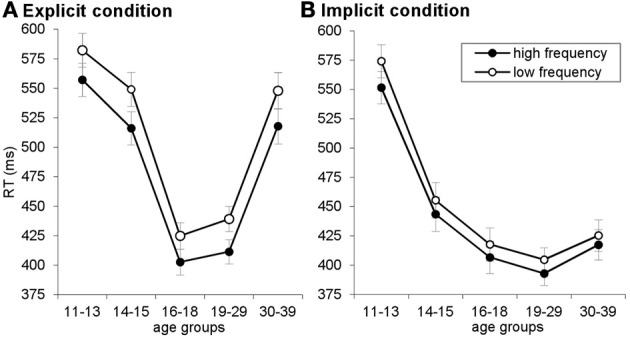
**Reaction time (RT) plotted for high- and low-frequency triplets, separately for the explicit (A) and implicit (B) conditions.** Error bars indicate standard error of mean (SEM).

Specifically, in the implicit condition, *post-hoc* tests revealed that the 11–13-years-old group exhibited the highest level of triplet learning, differing from all other groups (*p*s < 0.069) who performed on the same level between 14 and 39 years of age (*p*s > 0.408) (Figures 2, A1). In contrast, in the explicit condition, all age groups reached similar extent of triplet learning (*p*s > 0.147). Although there was a small advantage in the 14–15-years-old group, this was significantly higher only to the 16–18-years-old group's performance (*p* = 0.037). Comparing the extent of learning in explicit vs. implicit conditions separately for each age group, the *post-hoc* tests revealed similar level of triplet learning in the implicit and explicit conditions for the 11–13-years-old group (22.53 vs. 25.15, respectively, *p* = 0.644). In contrast, other age groups demonstrated higher triplet learning in the explicit condition than in the implicit one (*p*s < 0.033).

A similar ANOVA was conducted for the six *probe blocks*. The ANOVA revealed significant triplet learning [indicated by the significant main effect of TRIPLET: *F*_(1, 278)_ = 96.958, η^2^_*p*_ = 0.259, *p* < 0.001] such that RTs were faster on high than on low frequency triplets (Figure A3A). Neither the conditions nor the age groups showed differences in the amount of learning (*p*s > 0.248). In sum, this measure revealed that, on average explicit learning of sequences has an advantage over implicit learning at all ages with the exception of the 11–13-years-old-group.

### Explicit knowledge across the age groups and conditions

**Figure 3:**
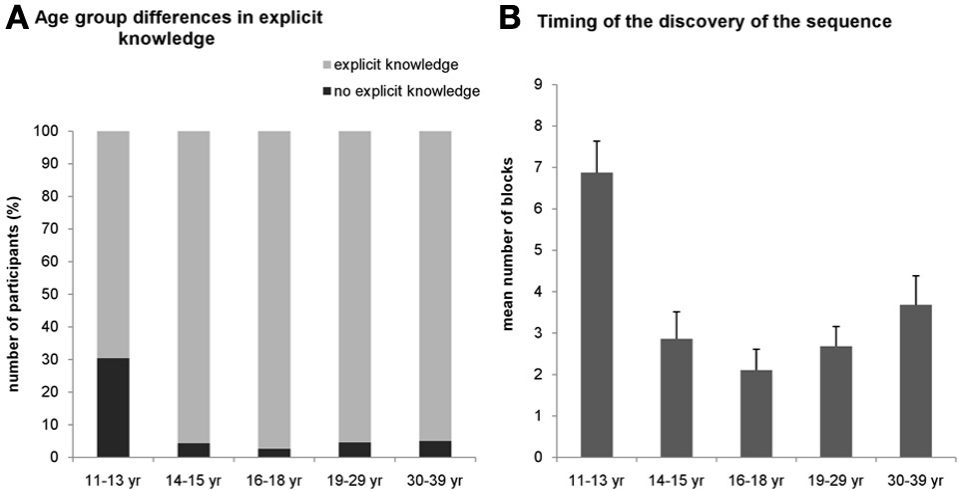
**Results of the verbal reports about the sequence knowledge in the explicit condition. (A)** Around 30 percent of the participants in the 11–13-years old group could not report the sequence throughout the whole task (black portion of bars) while this percentage was significantly smaller (~5%) in all other groups. **(B)** Participants in the youngest group who could report the sequence correctly gained this explicit knowledge significantly later (around the 6–7th block) than the older groups. Error bars represent standard error of mean (s.e.m.).

For the explicit condition, we assessed the number of participants in all age groups who gained explicit knowledge about the sequence during the task. The χ^2^-test revealed a significantly different distribution across age groups [χ^2^_(4)_ = 18.19, *p* = 0.001]. In the 11–13-years-old group, only 69.6% of the participants could report the correct sequence structure during the task while in other age groups, at least 95% of the participants gained explicit knowledge about the sequence (Figure 3A).

To further characterize the age differences in the explicit knowledge, we compared when subjects gained their explicit knowledge about the sequence during the experiment. We measured the number of the block where the participants could report the sequence structure consistently (i.e., reported the same correct sequence in all consecutive blocks). A univariate ANOVA (excluding those participants who did not succeed to report the correct sequence structure in the task at all) revealed significant difference among the age groups [*F*_(4, 130)_ = 7.440, η^2^_*p*_ = 0.186, *p* < 0.001] (Figure 3B). Specifically, the mean of the experimental blocks where the 11–13-years-old group reported the sequence consistently was 6.875, significantly differing from all other age groups, typically remaining around 2.83 (*p*s < 0.003). All other age groups did not differ significantly from each other (*p*s > 0.07). For the implicit condition, we did not ask participants to report the sequence after any block because it would have drawn their attention to the hidden structure of the task eliminating the implicitness of this condition. Instead, we collected a verbal report after the subjects finished the entire experiment, and found that none of them could report the correct sequence structure.

### Statistical and higher-order sequence learning across age groups and conditions

To further dissect the nature of learning among age groups and conditions, we conducted a mixed design ANOVA for *experimental blocks* with TYPE (3: pattern, random-high frequency and random-low frequency elements) and BLOCK (1–14) as within-subject factors, and AGE GROUP (11–13, 14–15, 16–18, 19–29, and 30–39 years of age) and CONDITION (explicit vs. implicit) as between-subjects factors. This ANOVA allowed us to disentangle a number of relevant factors influencing sequence learning.

**Figure 4:**
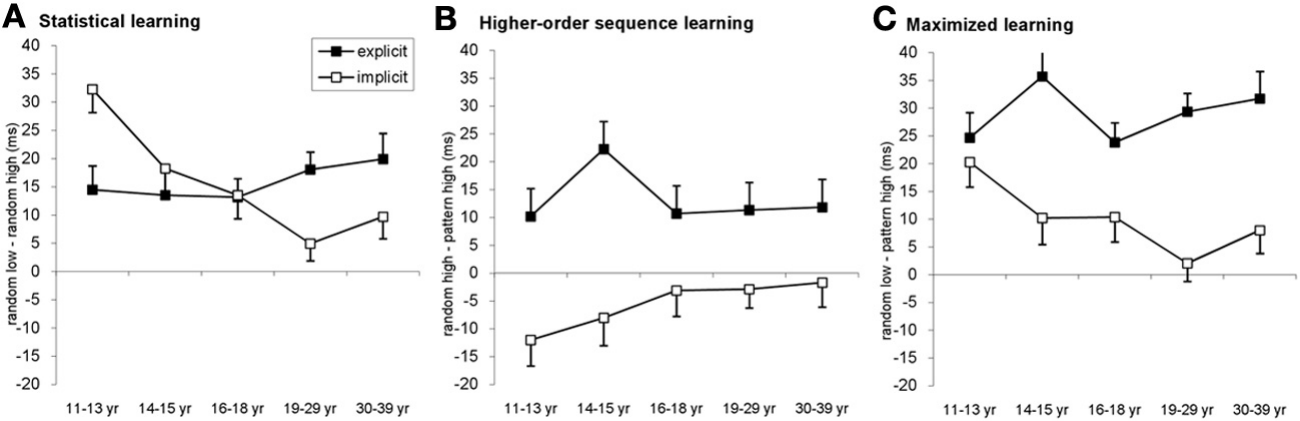
**Detailed analysis of the learning across age groups and conditions. (A)** Statistical learning (RT difference between random low and random high-frequency triplets) resulted in a gradually declining developmental curve in the implicit condition with an age invariant performance in the explicit condition. The youngest age group exhibited better statistical learning in the implicit condition compared to the explicit one while the opposite pattern was observable between 19 and 39 years of age. **(B)** In the case of higher-order sequence learning (RT difference between random high-frequency and patter elements) only groups in the explicit condition showed significant learning, with approximately similar extent of learning across ages. **(C)** The additive effect of the statistical and higher-order sequence learning is evident by maximized learning (RT difference between random low-frequency and pattern elements). The pattern is similar to the triplet learning results (cf. Figure 2): the highest learning of the 11–13-year olds in the implicit condition while mainly similar level of learning across age groups in the explicit condition. Error bars represent standard error of mean (s.e.m.).

**Figure A2:**
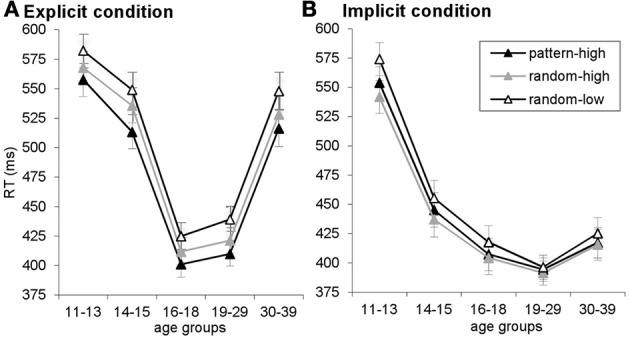
**RTs are plotted for pattern-high frequency, random-high frequency and random-low frequency triplets, separately for the explicit (A) and implicit (B) conditions.** Here, the difference between random-high- and random-low frequency triplets reflects statistical learning, the difference between pattern-high and random-high frequency triplets reflects higher-order sequence learning, and the difference between pattern-high and random-low frequency triplets reflects maximized learning. Error bars indicate SEM.

First, the analysis revealed a strong evidence for both statistical and higher-order sequence learning within the general learning effect of this task reported above (Figure 4). We found a significant main effect of TYPE [*F*_(2, 556)_ = 122.422, η^2^_*p*_ = 0.306, *p* < 0.001], suggesting that participants responded differently to pattern, random-high frequency and random-low frequency elements, respectively. Specifically, *post-hoc* test showed that they were the fastest on pattern elements (upper left cell Figure 1C; 461 ms), differing significantly both from random-high frequency (upper right cell, Figure 1C; 465 ms, *p* = 0.006) and random-low frequency elements (lower right cell, Figure 1C; 481 ms, *p* < 0.001). Thus, the RT differences between random-high and pattern-high triplets (higher-order sequence learning) as well as between random-low and random-high (statistical learning) were both significant (*p* < 0.001; Figure A2).

Second, we also found that the extent of these learning effects was different between the implicit and explicit conditions [significant TYPE × CONDITION interaction: *F*_(2, 556)_ = 33.511, η^2^_*p*_ = 0.108, *p* < 0.001]. In the explicit condition, participants exhibited significant higher-order sequence learning: responses for pattern elements were 13 ms faster than for random-high frequency elements (*p* < 0.001). In addition, they were significantly faster on random-high elements compared to random low elements, revealing statistical learning (15.81 ms, *p* < 0.001). The pattern vs. random-low difference was also significant (29 ms, *p* < 0.001). Surprisingly, in the implicit condition, participants were significantly *slower* on pattern elements compared to random-high elements despite the 4-fold difference in appearance frequency, thus demonstrating a *reversed* higher-order sequence learning (−5.5 ms, *p* = 0.005). The statistical learning was to a large extent similar to that in the explicit condition (*p* = 0.976), namely participants were 15.74 ms faster on random-high compared to random low elements (*p* < 0.001). Due to the reversed higher-order sequence learning, the pattern vs. random low difference (overall learning) in the implicit condition (10 ms, *p* < 0.001) was significantly smaller compared to the explicit condition (*p* < 0.001).

Third, investigating the effect of age within the ANOVA design added further detail to the emerging picture, as we found a significant TYPE × CONDITION × AGEGROUP interaction [*F*_(8, 556)_ = 2.936, η^2^_*p*_ = 0.041, *p* = 0.003]. Specifically, in the implicit condition, the 11–13-years-old group exhibited the highest level of statistical learning (Figure 4A), differing significantly from all other groups (*p*s < 0.02). The decline was monotonic and the drop was significant from level in the 14–15-years-old group to level in the 18–29-years-old group (*p* = 0.014). There was a notable lack of such monotonic decrement in learning under the explicit condition, where all age groups showed a similar magnitude of statistical learning (*p*s > 0.229), suggesting age invariance of such learning.

Comparing the extent of statistical learning in explicit vs. implicit conditions separately for each age group, the *post-hoc* tests revealed stronger statistical learning in the implicit condition than in the explicit one for the 11–13-years-old group (32.29 vs. 14.47 ms, respectively, *p* = 0.003) (Figure 4A). In contrast, the 19–29 and 30–39-years-old groups exhibited an opposite pattern by showing higher statistical learning in the explicit (18.05 and 19.90 ms, respectively) than in the implicit condition (4.9 and 9.7 ms, respectively). In the older age group, however, this difference did not reach significant (*p* = 0.003 for the 19–29-years-old group and *p* = 0.09 for the 30–39-years-old group). In the adolescent groups, the extent of the statistical learning was similar in both conditions (14–15-years-olds: 13.49 vs. 18.25 for explicit and implicit conditions, respectively, *p* = 0.438; 16–18-years-olds: 13.14 vs. 13.50, *p* = 0.947). Taken together, the difference between statistical learning in the explicit and implicit conditions reversed across age groups, children showing stronger learning in the implicit condition while adults demonstrating stronger learning in the explicit condition.

In the case of the higher-order sequence learning (Figure 4B), the effect of learning was stronger in the explicit than in the implicit condition (*p*s < 0.045 for all age groups). Analyzing the conditions separately, in the explicit condition, all age groups showed a significant learning effect (*p*s < 0.03). The extent of this learning was similar in all age groups, except for the 14–15-years-olds who performed slightly but not significantly better than the other groups (*p*s between 0.056 and 0.136). In the implicit condition, the RT difference between pattern and random-high elements was not significant between 16 and 39 years of age (*ps > 0.397)*, however, the 11–13-years-olds demonstrated a significant *reversed learning*, being faster on random-high elements compared to the pattern elements (*p* = 0.01), and the 14–15-years-old group showed a similar albeit non-significant trend (*p* = 0.106). Neither of these two groups differed significantly from the older groups in learning (*p*s > 0.107). Thus, summing up the different local patterns, we found (a) significant and quasi-age-independent advantage of the explicit condition over the implicit one, (b) a significant learning effect in the explicit condition across the board, and (c) a significant interference in the youngest subjects in the implicit condition.

We also conducted a correlational analysis to examine the relationship between verbal reports and RT learning measures. We found a significant correlation between the timing of the discovery of the sequence and the extent of higher-order sequence learning (*r* = −0.22, *p* = 0.01, corrected for age), such that the earlier the participants could report the sequence structure, the better their higher-order sequence learning performance was in the explicit condition. In contrast, there was no correlation between the verbal reports and the statistical learning measure (*r* < 0.1), suggesting that this type of learning is not related on explicit knowledge.

In order to assess the effect of all structures carried by the sequences on learning, one needs to compare the difference between the pattern-high and the random-low conditions (upper left and lower right cells in Figure 1C). This analysis provides a clear indication of the purely additive effect of statistical and higher-order sequence learning where different parts of the two curves (different age groups) are controlled more strongly by different types of learning (Figure 4C). For example, learning by the 11–13-years-olds is similar in explicit and implicit conditions (*p* = 0.49) and this similarity is determined mainly by larger statistical learning combined with a larger interference in the higher-order learning in the implicit condition compared to an average statistical and higher-order sequence learning in the explicit condition (Figures 4A,B). In the other age groups, the overall stronger maximal learning in the explicit condition (*p*s < 0.019), is driven by the advantage of the explicit condition in both statistical and higher-order sequence learning which is not compensated any more (in fact, enhanced) by the reduced statistical learning advantage and nonexistent learning in the higher-order learning in the implicit condition.

**Figure A3:**
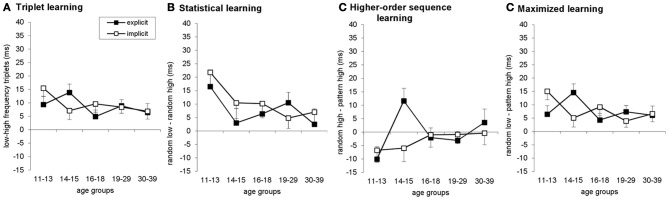
**Learning measures in probe blocks are plotted for triplet (A), statistical (B), higher-order sequence (C), and maximized learning (D).** Error bars indicate SEM.

To round up our analysis, a similar mixed-design ANOVA was conducted for the six probe blocks of the experiment (Figures A3B–D). This ANOVA revealed a significant main effect of TYPE [*F*_(2, 554)_ = 27.953, η^2^_*p*_ = 0.092, *p* < 0.001] due to the RTs with pattern and random high frequency triplets (459.95 ms vs. 458.44 ms, respectively) being significantly faster than that of random low frequency triplets (467.75 ms, *p*s < 0.001), with no difference between pattern and random high triplets (*p* = 0.274). Neither the Conditions, nor the Age group × Condition interaction reached significance (*p*s > 0.207).

### Within-block effects on learning across age groups and conditions

**Figure 5:**
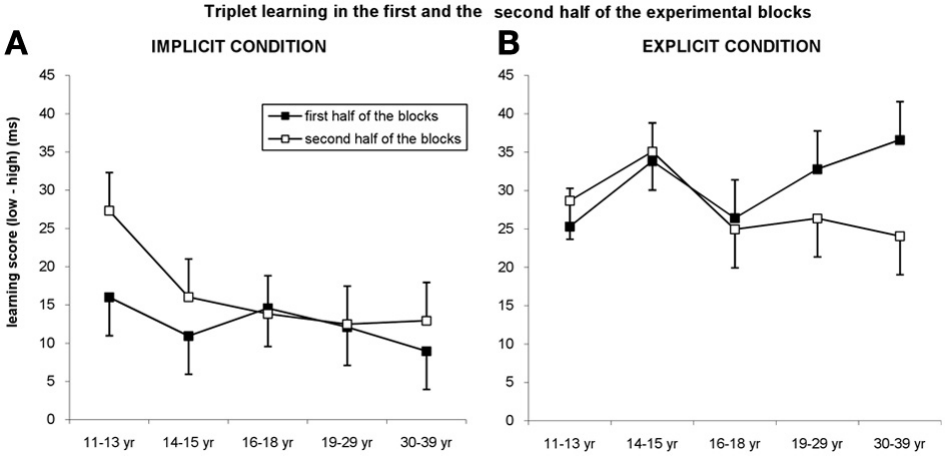
**Triplet learning in the first and second half of the blocks. (A)** In the implicit condition, the youngest group outperformed the other groups in the second half of the blocks, while they exhibited a similar level of learning in the first half of the blocks. **(B)** In the explicit condition, participants between 19 and 39 years of age showed higher learning in the first half of the blocks compared to the second half while other groups exhibited the same level in both parts of blocks. Error bars represent standard error of mean (s.e.m.).

We further analyzed our data by splitting each block into two halves, to investigate earlier claims that reactive inhibition emerges within blocks, masking the potential learning effects (Rickard et al., 2008; Brawn et al., 2010). According to these reports, the longer people have to perform a reaction time task arranged in blocks of, for example, several seconds or minutes, the slower they become by the end of each block, and consequently, their performance is the best at the beginning of each block (Rickard et al., 2008; Brawn et al., 2010). Since younger children can be more affected by this kind of fatigue/slow-down, it is important to take this effect into account when comparing learning performances across a wide range of ages. Therefore, we conducted a mixed design ANOVA on *experimental blocks* with TRIPLET (high vs. low frequency), BLOCK (1–14), and PART (first vs. second half of blocks) as within-subject factors and AGE GROUP (11–13, 14–15, 16–18, 19–29, and 30–39 years of age) and CONDITION (explicit vs. implicit) as between-subject factors (Figures 5A,B).

The ANOVA revealed significant triplet learning overall [main effect of TRIPLET: *F*_(1, 278)_ = 312.945, η^2^_*p*_ = 0.53, *p* < 0.001], with higher learning for the explicit condition compared to the implicit one [29 vs. 14 ms; TRIPLET × CONDITION interaction: *F*_(1, 278)_ = 35.997, η^2^_*p*_ = 0.115, *p* < 0.001]. Interestingly, taking the PART of the blocks into account, we found a significant TRIPLET × CONDITION × PART interaction [*F*_(1, 278)_ = 7.539, η^2^_*p*_ = 0.026, *p* = 0.006]: triplet learning was greater in the second part of the blocks compared to the first part for the implicit condition (12.5 vs. 16.5 ms, *p* = 0.032), while the opposite trend was obtained for the explicit condition (30.98 vs. 27.81 ms, *p* = 0.086) (Figure 5). Although the TRIPLET × CONDITION × PART × AGE GROUP interaction did not reach significance [*F*_(4, 278)_ = 0.962, η^2^_*p*_ = 0.014, *p* = 0.429], planned comparisons revealed that in the 11–13-years-old group, the triplet learning was greater in the second part of the blocks (27.30 ms) compared to the first part of the blocks (15.98 ms) but only in the implicit condition (*p* = 0.01). By contrast, in the explicit condition the extent of learning was similar in both parts of the block (25.30 vs. 28.66 ms, *p* = 0.449). The opposite pattern was observed between 19 and 39 years of age: the extent of learning in the first and second half of blocks was similar in the implicit condition (*p*s > 0.33), while they exhibited greater learning in the first half of blocks than in the second half in the explicit condition (19–29-years old group: 32.78 vs. 26.36, *p* = 0.049; 30–39-years old group: 36.59 vs. 24.04 ms, *p* = 0.009). Between 14 and 18 years of age participants showed similar extent of learning in both parts of the blocks in both conditions (*p*s > 0.275).

A similar ANOVA was conducted for the six *probe blocks*. The ANOVA revealed significant main effect of TRIPLET [*F*_(1, 278)_ = 147.602, η^2^_*p*_ = 0.347, *p* < 0.001] such that RTs were faster on high than on low frequency triplets. The TRIPLET × PART × AGE GROUP was marginally significant [*F*_(4, 278)_ = 2.377, η^2^_*p*_ = 0.033, *p* = 0.052]. *Post-hoc* tests revealed that the 11–13-years old group exhibited larger triplet learning in the first part of the blocks (21.08 vs. 12.91 ms, *p* = 0.039) collapsed across conditions, while the opposite trend was observable in the 14–15- and 19–29-years old groups (*p*s < 0.09). Other interactions regarding the CONDITION and AGE GROUP were not significant (*p*s > 0.373).

## Discussion

There were two main aims of the present study. First, we wanted to obtain a detailed and systematic description of probabilistic sequence learning in both explicit and implicit setups so that this kind of skill-related learning could be related to other types of purely perceptual learning domains such as visual statistical learning (Fiser and Aslin, 2001, 2002, 2005). Second, using the insights of the first aim, we wanted to test the hypothesis that there is a coherent shift in the interaction between simple raw probability-based and complex, internal-model based learning at around the age of 13 (Janacsek et al., 2012). To this end, we investigated the differences of explicit and implicit probabilistic sequence learning in different age groups between 11 and 39 years of age.

To fulfill the first aim, we analyzed pure statistical vs. higher-order sequence learning separately. In the case of classical triplet analysis used in many previous studies (Howard and Howard, 1997; Song et al., 2007a; Nemeth and Janacsek, 2011; Janacsek et al., 2012), the triplet frequency information (high vs. low frequency elements) was mixed with sequence information (random vs. pattern elements), making the interpretation difficult and fuzzy. With our new analysis methods, we could factorize the problem of triplet learning and clarify the nature of the underlying learning mechanisms. We quantified pure statistical learning as the difference in reaction time to high and low probability random events (random-low minus random-high frequency triplets, Figure 1D) independent of the long-range sequence information. In contrast, we measured higher-order sequence learning by RTs capturing the difference between the ability to internalize a triplet on its own vs. the same triplet embedded in a repetitive larger structure (random-high minus pattern-high frequency triplets, Figure 1D) while the simple statistical information about the elements within the triplets is equated. Hence our *statistical learning* is a measure of acquiring knowledge of the local statistical structures (individual appearance probabilities), while higher-order sequence learning is a measure of becoming sensitive to long-range relational structures. Both type of learning is based on the input statistics, but they measure independent aspects of the input structure, and while simple local structures are presumably easier to learn right away, learning global structures might get a serious boost from additional ability to handle more complex memory constructs, which we refer to as the internal models utilized in model-based learning. We also propose that our higher-order sequence learning measure is more closely related to explicit knowledge that is more suited for explicit learning. This proposal has been corroborated by our finding of a significant correlation between the timing of the discovery of the sequence and the extent of higher-order sequence learning in the explicit condition. Notice that no such correlation was detected for statistical learning supporting our view that the simple statistical relationships discovered by this type of learning are more readily subject of model-free learning.

Our first important finding is related to the fact that explicit learning seriously boosts the ability to learn higher-order structures by directly focusing the subjects' attention on the relevant structures in our task (Figure 4B). Within this analysis, we also found that in the implicit learning setup, learning higher-order structures by younger children is significantly interfered with whereas in older subjects the effect of higher-order structures is approximately zero (Figure 4B). This provides our first hint that a significant gradual shift occurs in the processing of more complex information of the input around the age of 13, which can be detected in an implicit task.

In the case of *statistical learning*, we found a gradual decline across ages in the implicit condition, contrasting the age invariant learning effect we measured in the explicit condition (Figure 4A). However, the performance in the explicit condition was inferior to the implicit case around 11–13 years, while it was better beyond the age of 19. It is important to consider two facts in interpreting these results. First, the flat explicit developmental curve does not mean that subjects would perform invariantly in ANY kind of explicit sequential task, only that in the present task, the complex interaction between explicit and implicit processes result in a fairly constant performance. Second, since our explicit measure always combines explicit and implicit learning (i.e., there is no purely explicit learning), the comparison of the implicit and explicit results should always be interpreted in a relative manner, that is how much the explicit learning machinery adds or interferes with the basic implicit learning processes. Thus, while due to their independence, statistical, and higher-order sequence learning results can be considered separately and combined additively to obtain the results of maximized learning both for implicit and explicit learning separately (see Figure 4C), the same kind of independent treatment cannot be applied between implicit and explicit results of either type of (statistical or higher-order sequence) learning. Specifically, the flat explicit learning performance during statistical learning (Figure 4A) is not an indicator of unchanging ability of extracting explicit knowledge-based information at different ages.

With these two points in mind, our interpretation of the above statistical learning results (Figure 4A) is that despite the steady decrease of implicit performance with age, subjects manage to keep the overall performance at older ages - as measured in the explicit task- from falling, presumably with the increasing help of learning processes evoke by the explicit information. In other words, young children could pick up raw probability information better if no explicit influence interfered with their implicit processes, whereas this implicit learning ability deteriorated with age but also received a serious boost from explicit-knowledge-based help when the subject was more mature. We propose that the performance in implicit statistical learning is more directly related to the model-free processes mentioned in the introduction, while the addition of explicit information leading to interference in young age and boost in older age in the explicit learning task is related to the contribution of the model-based learning processes that can more effectively extract higher order structures.

The analysis of the explicit knowledge about the sequence structure also supports the idea that the interaction between model-free and model-based processes can explain the pattern of the implicit and explicit learning results: we found that the 11–13 years old group gained explicit knowledge of the higher-order structures slower and less effectively compared to later ages (Figure 3). Specifically, these results demonstrate that the relationship between model-free and model-based processes (also termed sometimes as the implicit and explicit processes) is of a competitive nature (Poldrack et al., 2001): the less knowledge acquired explicitly on the structure the more implicit learning effect we have.

Returning to *higher-order sequence learning* in the explicit condition, there was a strange peak at ages 14 and 15 around the age where the reversed learning effect appeared in the implicit condition (Figure 4B). We speculate that these effects might be related to the gradual shift in dominance between purely local statistical and more global higher-order learning suggesting that the underlying computational mechanisms of the two types of learning use fundamentally different and somewhat complementary components. Specifically, at younger age, even higher-order relations are detected with a superior ability to extract raw probability structures, while around the age of 13, the same performance starts to be achieved by a very different strategy relying more on the utilization of explicitly treatable information.

In general, our result and their interpretation provides a very different and more complex picture about the development of human sequence learning compared to the earlier developmental proposals based on age-invariance (Meulemans et al., 1998; Vinter and Perruchet, 2000) or the inverted-U shape curve (Maybery et al., 1995; Fletcher et al., 2000; Thomas et al., 2004). We propose that (a) there are multiple learning processes playing parts in sequence learning, namely model-free and model-based learning, (b) in simple model-free learning tasks based on raw probabilities of events, young children are superior compared to adults, (c) for learning more complex types of patterns, model-based learning develops somewhat later at around 13 years of age, (d) incorporating model-based features into overall learning interferes, by definition, with the superior sensitivity to raw probabilities of model-free learning, and (e) nevertheless, the overall ability to learn all sorts of tasks in our environment improves with the integration of the model-based learning component.

To understand more thoroughly the developmental curve of sequence acquisition, it is worthwhile to consider memory processes such as reactivation and reconsolidation in these types of tasks (Walker et al., 2003; Rickard et al., 2008). During the acquisition of sequences we are learning, recalling, and reactivating the sequence elements continuously. Recalling or reactivating a previously consolidated memory makes it fragile and susceptible to interference once again, therefore requiring periods of reconsolidation (Walker et al., 2003). These repetitions of the recall, reactivation, and consolidation processes allow a continuing refinement and reshaping of previously learned motor or cognitive skills in the context of ongoing experience. In experimental designs (fingertapping or SRT tasks) and partly in real-life situations, we are learning sequences arranged in blocks, which are separated by shorter or longer time periods. Several recent studies showed that the separate analysis of the different parts of the learning blocks is crucial in understanding the consolidation and reconsolidation of sequence learning (Rickard et al., 2008; Brawn et al., 2010; Nemeth et al., 2013b). In particular, in the beginning of the blocks we have to recall and reactivate the sequence structure partly learnt already in the previous blocks. The second part of each block might be responsible for the reconsolidation of the sequence structure. In our study, we found that this “detection of probabilities - reactivation/recall—reconsolidation” cycle is different across ages and conditions: while in the implicit condition the learning of the second half of the blocks is better than the learning in the first half of the blocks in younger ages, in the explicit condition an opposite pattern emerged with better performance in the first half of the blocks in older ages (Figure 5). These results suggest that the memory reactivation processes are weaker before early adolescence in the implicit condition, presumably because of the weaker model-based processes. However, when subjects have an efficient cue to find the hidden structure in the explicit condition, it can boost the model-based processes as reflected in the similar extent of learning in the first and second half of the blocks, but only after 16 years of age. In older ages the reactivation of the previously acquired knowledge is more effective in the explicit condition, with a weaker performance in the second half of the blocks (Figure 5B). These results can be connected to fatigue effect caused by a more attention demanding explicit learning because this effect disappears in the implicit condition where cognitively controlled processes are less dominant.

Although we focused on experimental blocks in this study, we also administered probe blocks in order to investigate the transfer of the acquired knowledge from the more controlled learning situation to a more automatic one. Our results showed that although all groups exhibited learning in these probe blocks, the gain of explicit instructions diminished in most cases, suggesting that this amount of learning is not enough to build a deeper representation about the sequence structure. In other words, in spite of whether or not the participants were able to form some type of internal representation of the sequence structure, this learning was not enough to generate an automatic, procedural behavior. This could be the reason for failing to find developmental differences in these probe blocks.

To sum up, the present study provides additional support for the developmental framework proposed in Janacsek et al.'s (2012) study: there is a shift in early adolescence when the system adapts efficiently to more complex aspects of the world by relying more on internal model-based interpretations, while somewhat neglecting the raw probabilities of the sensory input. The results also corroborates the findings that the cortical areas implied in storing the internal models for model-based learning become truly functional late in the development, around early adolescence (12–14 years of age; Giedd et al., 1999; Blakemore and Choudhury, 2006). In addition, by separating the different components of sequence learning, our results could also demonstrate the competitive interaction between simple model-free and more complex model-based memory processes (Poldrack et al., 2001; Logothetis et al., 2012; Nemeth et al., 2013a). Finally, these results build a bridge between the classical domain of procedural skill learning and the more perceptual-type statistical learning literature raising the possibility that despite obvious differences, these processes share partially the same computational bases.

### Conflict of interest statement

The authors declare that the research was conducted in the absence of any commercial or financial relationships that could be construed as a potential conflict of interest.
